# Sexual Orientation and Infidelity-Related Behaviors on Social Media Sites

**DOI:** 10.3390/ijerph192315659

**Published:** 2022-11-25

**Authors:** Ionela Șerban, Marco Salvati, Violeta Enea

**Affiliations:** 1 Department of Psychology, Alexandru Ioan Cuza University, 700554 Iaşi, Romania; 2 Department of Human Sciences, University of Verona, 37126 Verona, Italy

**Keywords:** gay, lesbian, same-sex couples, online infidelity, dyadic satisfaction

## Abstract

Little research has been focused on offline or online infidelity in GL dating relationships, especially in a post-communist socio-cultural context. Infidelity–related (IR) behaviors on social media sites might be as hurtful to relationships as offline infidelity, both in gay, lesbian (GL) and heterosexual romantic monogamous relationships. In this cross-sectional study, we aimed to examine the associations between dyadic satisfaction, attitudes toward infidelity, and problematic internet usage, with IR behaviors on social media sites among GL and heterosexual unmarried individuals in Romanian sexual minority communities. Results showed that GL respondents did not significantly differ from heterosexual participants regarding IR behaviors. Furthermore, we found the main effect of attitudes toward infidelity and problematic internet use on IR behaviors. Sexual orientation highlights the main effect of IR behaviors when analyzed with dyadic satisfaction. The current study may be a precursor to further research investigating correlations in online IR behavior among lesbian and gay individuals engaging in consensual nonmonogamy. Implications of the findings are discussed in the social context of a post-communist country where GL individuals may face discrimination and stigma because of their sexual orientation.

## 1. Introduction

The internet provides a favorable environment for the development of all the sub-processes that underlie socialization, such as communication, association, identification, culturalization, and differentiation. This characteristic makes the internet space a socializing agent as important as face-to-face interactions [1]. Digital communication through social media sites can create social connectedness through the sense of belonging and relationships that it offers [2]. Social networks are designed to facilitate interaction between people in the virtual environment [3]. Some researchers have defined them as services that allow people to build a public or semi-public profile in a limited system, to build a list of other users they want to connect with, to see and analyze their friends, as well as others, in this system [4].

According to Facebook Statistics, this network represents the largest social media platform and has 2.93 billion active monthly users all over the world (July 2022). Facebook facilitates the creation of close relationships more easily than face-to-face interaction. Unlike face-to-face interaction, in which initial reluctance appears when sharing personal information until the relationship is familiar enough, Facebook offers access to all information on the user’s profile, including personal information, referring to interests, opinions, favorite activities, and preferences such as movies, music, or politics [5].

A growing body of research has suggested that Facebook usage predicts negative relationship outcomes, such as cheating, breakups, and divorce [6]. Thus, social networks provide a way to initiate and perpetuate behaviors that can be potentially harmful to romantic relationships [5]. In other words, social media platforms could be used for infidelity behaviors, and researchers have reported that these online platforms are often used for that [5]. The most common behaviors related to infidelity on Facebook were reported as befriending a former partner, sending private messages to an ex-partner, adding comments to photos of attractive people, and posting an inaccurate relationship status [6]. Specifically, internet infidelity represents a romantic or sexual relationship with a person other than your official partner, initiated on the internet and supported by technology [7]. 

Romania is one of the Eastern European countries where the rights of the sexual minority are still discussed [8]. From a legal point of view, marriage and child adoption are not allowed, with citizens highlighting their preference for traditional family practices [9]. According to the European Value Study in which Romania participated in 2008, it seems that Romanian citizens have negative attitudes towards sexual minority individuals and tend to consider their behavior as morally wrong [8]. In a study conducted by a Romanian association (ACCEPT) to explore the sexual minority community in Romania, 68.2% of participants indicated that they had experienced discrimination and exclusion; meanwhile, 30% had experienced physical violence because of their sexual orientation [10]. 

Sexual minority community characteristics are poorly analyzed in the post-communist socio-cultural context of Eastern European countries like Romania. Therefore, our study is the first to specifically explore the relationship between sexual orientation and IR behaviors on social media sites, including a series of demographic factors and interpersonal variables in the analysis of Romanian GL individuals. The current study focuses on analyzing the relationships between attitudes toward infidelity, internet use, and couple satisfaction with IR behaviors on social media sites among unmarried GL and heterosexual individuals.

### 1.1. Theoretical Framework: The Ecological Perspective of Couple and Family Technology Framework

The Couple and Family Technology Framework [11] suggests that the characteristics of the internet and technology may both positively and negatively impact a couple’s relationship in terms of initiation, maintenance, and termination. The model is composed of three domains and proposes that ecological elements of technology (1) produce changes in couple structure (2) and processes (3) [12].

The ecological perspective of this model postulates that technology, by its three characteristics (accessibility, affordability, and anonymity, also called the “Triple-A engine” proposed by Cooper [13]) cause changes in the structure of the relationship by redefining roles, boundaries, and rules. Accessibility represents the ability to easily meet a new or potential partner, affordability refers to the low cost provided by the internet associated with meeting new people, and anonymity is the ability to hide one’s identity or to remain discreet [14]. However, according to McKie et al. [14], these characteristics of the internet have been improved by adding acceptability (higher levels of online tolerance) [15], approximation (which refers to the questionable nature of the truth in the online environment) [16], and accommodation (the relationship between the real and ideal self in an online environment, and ambiguity) [11]. Furthermore, for gay men, this model is further validated by adding assessment and affirmation [17]. Assessment refers to the ability to assess compatibility with a potential partner, and affirmation represents one’s capacity to explore their own identity [14]. The changes that ecological factors bring to the relationship process are related to redefining intimacy, initiating romantic relationships, and maintaining a romantic relationship. Structure and process changes in couples produced by ecological factors are also linked to one another. Therefore, transformations in the structure of relationships can produce changes in the processes of relationships, and vice versa.

Regarding the ecological perspective of the Couple and Family Technology Framework [11], we postulate that internet use, attitudes toward infidelity, and dyadic satisfaction may influence IR behaviors on social media sites, which is a relationship process change related to relationship initiation and formation processes. According to this model, one of the main differences between face-to-face communication and computer-mediated communication is that people attribute certain characteristics to their online partner, and in some cases, those characteristics may not be accurate [11]. In this situation, the idealization of the partner and the risk that a romantic relationship will be based on fantasy rather than reality can appear [11,18].

### 1.2. Online Infidelity-Related (IR) Behaviors and Attitudes toward Infidelity

The online environment represents a potential space for meeting potential sexual partners, in part due to its key features: affordability, access, and anonymity [19,20]. For example, the anonymity offered by the internet allows young gay men to communicate more openly than in a face-to-face social interaction situation [21]. Through online communication, technology facilitates finding partners available for sexual or romantic relationships, maintaining relationships, and also ending relationships, among young gay men [14]. 

Infidelity can be defined only depending on each couple’s rules and boundaries. Some authors have defined infidelity as sexual or/and emotional behavior with another person exhibited by someone already in a committed relationship, and this behavior violates the agreement regarding sexual and/or emotional exclusivity in the romantic relationship [22]. In addition, the relational context is extremely important when defining infidelity. For example, a monogamous relationship may consider a personal or emotional relationship with an extradyadic person as infidelity. However, multiple partners are not considered to constitute infidelity in consensual nonmonogamy (e.g., open relationships, polyamory) [23]. Research has suggested that, in order to define infidelity in same-sex relationships, it is important to consider the consensuality between partners regarding extradyadic behaviors, the type of relationship, and the types of behaviors that may constitute infidelity [24]. Recently, Lehmiller and Selterman [24] found that both consensual nonmonogamy and nonconsensual nonmonogamy were more prevalent among sexual minorities. 

Online infidelity-related behaviors (IR) are those behaviors that unfaithful people may engage in, such as feelings of discomfort in imagining your official partner reading online messages sent or received, hiding information or secrets from the partner, forming emotional connections with a person other than the official partner, looking for ex-partners or defensive behavior regarding the suspicion of infidelity [5]. Empirical literature related to problematic IR behaviors on social media sites (e.g., befriending attractive alternative partners, engaging in cybersex) and relationships between heterosexual young adults and married/cohabiting individuals have expanded over the past few years [5,25,26,27]. However, IR behaviors on social media sites have been rarely studied in GL individuals. In a review study that closely investigated the romantic relationships of GL people, the results showed that, of the GL people who engaged in love affairs outside the couple, gay men reported the highest number of sexual partners [28]. Researchers reported that gay men are more likely than heterosexual men to use technology in order to meet potential partners and, from the total amount of same-sex couples participating in a study, nearly 70% of them reported meeting each other online [29]. One study that examined the factors associated with IR behaviors on social media sites in GL unmarried individuals found that technology is a challenge for young sexual minority individuals in terms of maintaining a romantic relationship [14]. The interviewees reported the difficulty of maintaining loyalty to the partner in the context of increasing requests, but also in terms of access to a new partner [14].

Some people who spend too much time on the internet are involved in an online romantic relationship, even though they have an offline partner [30,31]. The high level of use of Facebook can be detrimental to interpersonal relationships, and people who spend more time on Facebook neglect their partner by reducing time spent together. Communicating with other people or former partners and other similar behaviors can lead to conflict, or a breakdown of the relationship [6]. The negative effects of online romances also include neglect of work and less interest in sex with the official partner, with almost two-thirds of the participants reporting they had sex with their internet partners [31].

Regarding attitudes toward infidelity, there is a greater likelihood that people with liberal attitudes concerning sex and sexuality will assert the existence of an extra-conjugal relationship [32]. Moreover, previous research results indicated that attitudes toward infidelity may depend on specific behaviors that translate into betrayal [33]. Betrayal is considered acceptable if the relationship was already going to deteriorate, but less acceptable if engagement in unfaithful behavior has been planned or performed as a form of revenge [34,35]. The members of a couple often interpret daily interactions with another person other than the partner as acceptable if the possibility of a sexual act is low or absent, because sustained physical interaction is a precursor to sexual behaviors [35,36]. 

### 1.3. Dyadic Satisfaction

Researchers in the field have postulated that dyadic satisfaction represents a person’s overall assessment of their own romantic relationship, and there are some indicators that measure dyadic satisfaction and assesses how different aspects of a relationship are related to individual partner functioning [37,38]. According to Spanier [39], dyadic satisfaction is a concept that has several components, such as dyadic consensus (the level of agreement on matters important to the relationship such as religion, recreation, friends, house tasks, and time spent together), dyadic satisfaction (which represents the amount of tension in the relationship and the extent to which partners have considered ending it), affectional expression (the level of satisfaction regarding affection and sex in the relationship), and dyadic cohesion (interests and activities that the two partners have in common). Another perspective is offered by a model derived from the interdependence theory [40]. According to this model, a person is satisfied with their romantic relationship if he/she perceives many benefits from the relationship, with few costs to being in the relationship, and evaluates the relationship as exceeding an optimal standard of what a good relationship means [40].

Studies have found that a couple’s level of relationship satisfaction can predict if the partners will remain together or if they will break up [41], because individuals who are more satisfied with their relationship tend to be more committed and invest more resources in that romantic relationship [42]. Young people have specified dyadic dissatisfaction as a significant reason for engaging in infidelity behaviors [31,43]. Perceived dissatisfaction with the relationship is a causal factor in a person’s involvement in unfaithful behaviors because, at a time of sadness, anger, or frustration in a relationship, a person may detach with the help of another person who provides empathy and support [44].

Internet and social media sites might have a huge impact on dyadic satisfaction. Valenzuela et al. [45] found that greater use of Facebook predicted lower levels of marital satisfaction and a higher incidence of divorce. They concluded that an online environment can provide social support to those who have unhappy marriages, providing opportunities to engage in infidelity behaviors that can cause conflict and erode marital quality [45].

### 1.4. Same-Sex Couples’ Well-Being and Satisfaction

On the one hand, same-sex couples share several aspects with heterosexual couples [46,47]. On the other hand, they have to face specific challenges [48] due to heteronormative society, discrimination, minority stress, intersectionality, lack of family, social, and institutional support, and potential agreements around extradyadic sex [49,50]. All these characteristics contribute to making them a high-risk group to experience increased vulnerability in terms of their relationship and social wellbeing [51,52]. Indeed, same-sex couples are more likely to encounter both general life stressors and minority stressors, which are related to being members of a stigmatized group in society [53,54]. Specifically, there are both distal and proximal minority stressors which can include external heterosexist discrimination, expectations of heterosexist discrimination that derive from the appraisal of the environment as threatening, and the internalized sexual stigma, which consists of the internalization of negative societal attitudes toward sexual minority people [55,56,57]. 

Internalized sexual stigma is associated with low self-esteem, fears of intimacy, attachment insecurity, and doubts about oneself and one’s partner, which are all risk factors for couple relationship wellbeing. Furthermore, same-sex couples with high internalized sexual stigma tend to endorse stereotypical negative views of same-sex relationships, such as the fact that they are unstable with low investment, making them more inclined to avoid long-term relationships with high levels of commitment [57,58]. Another factor implicated in the wellbeing of a couple’s relationship is the question of visibility, also linked to a heteronormative and heterosexist society. Individuals in a same-sex couple who did not come out might dedicate time and energy to managing the visibility of their sexual orientation, subtracting such resources that could be spent on the wellbeing of the couple [58]. Romania is one of the most homophobic countries in the EU based on legislation and hate speech against LGBT individuals [59,60].

Few studies have investigated dyadic relationships by GL individuals. These individuals positively evaluate their relationship and describe it as stable and happy [61]. Other researchers have reported that individuals in married GL couples may be more likely to end the relationship when they perceive an alternative to the relationship to be more desirable [62]. In a longitudinal study conducted on GL individuals, results showed that partners who left a romantic relationship reported high romantic relationship dissatisfaction and invested few personal resources in the relationship, such as time or financial and emotional investment [63].

Satisfaction in romantic relationships follows the same line for GL individuals as for heterosexual individuals [63]. Relative to heterosexual couples, same-sex couples tend to experience similar levels of relationship satisfaction [46]. In 50% of the comparisons, GL individuals did not differ from heterosexual individuals in terms of domains indicative of relationship health, like personality traits, conflict resolution, and social support [64]. Regardless of sexual orientation, individuals whose level of relationship satisfaction increased see their partners as a source of support and comfort, and have a greater desire to express their warmth and affection for their partner [63].

A difference between GL and heterosexual individuals can be noted in the levels of jealousy, since non-GL participants reported higher levels of sexual jealousy compared to GL participants [65]. Specifically, heterosexual women scored higher in reactive jealousy, virtual jealousy, and self-reported jealousy compared to heterosexual men and GL individuals [66], whereas lesbian women reported lower levels of online jealousy compared to heterosexual women [66,67]. The researchers concluded that, for GL people, the status of being in a relationship is associated with less upset over sexual infidelity [68].

A high level of Facebook usage may be harmful to romantic relationships [6], predicting lower levels of marital satisfaction and offering opportunities to engage in infidelity-related behaviors online [45]. Furthermore, people with low dyadic satisfaction are much more inclined to engage in online interactions, while these interactions can cause a decrease in dyadic satisfaction [5].

### 1.5. Overview of This Study

A very recent systematic review of the literature that addressed factors associated with offline infidelity highlighted its complexity and indicated the impact of demographic factors and sexual information on infidelity [69]. To expand on the prior work, we examined the association of some different individual and interpersonal factors with online IR behaviors in GL and heterosexual individuals. The aims of the current study were to investigate: (1) whether the levels of engaging in IR behaviors on social media sites, attitudes toward infidelity, problematic internet usage, and dyadic satisfaction differ as a function of sexual orientation, and (2) whether engaging in IR behaviors on social media sites is related to attitudes toward infidelity, problematic internet usage, and dyadic satisfaction among unmarried GL and heterosexual individuals.

Based on the available evidence, we hypothesized that: (1) GL individuals report similar levels of IR behaviors compared with heterosexual individuals; and (2) attitudes towards infidelity, the level of problematic internet usage, and dyadic satisfaction will influence IR behaviors on social media sites among heterosexual individuals. Given the lack of studies examining the association between online IR behaviors and all these variables in GL individuals, we conducted an exploratory investigation into online IR behaviors and GL individuals in a monogamous relationship. Because of their apparent importance, the present study includes a number of variables as covariates (gender, age, the relationship duration, and time spent on social media sites).

## 2. Method

### 2.1. Participants and Procedure

Ethical approval for the study was obtained from the Ethics Committee of the Faculty of Psychology and Education Sciences. The questionnaire was administered over the internet, on Facebook, including groups for GLBT individuals (GLBT We Love, etc.). After opening the link, a short description of the study was given and participants had to complete an informed consent form, in which anonymity and voluntary participation were highlighted. The average time for completing the questionnaire form was around 20 min. Participants were not compensated for their participation.

An initial count of 156 individuals who had a personal profile on Facebook, Instagram, and Snapchat accessed the survey. Of this group, 10 bisexual individuals (5 men and 5 women), due to small numbers for comparison, 12 women with heterosexual orientation who did not completely fill in the questionnaire, and 13 married participants were excluded from the study. Thus, the final sample consisted of 121 unmarried participants aged 18–60 years (M age = 24.01, *SD* = 5.89), of which 81 identified as heterosexual (35 men and 46 women) and 40 identified as sexual minority individuals (30 gay and 10 lesbian individuals). Sociodemographic characteristics are presented in Table 1.

### 2.2. Measures

A demographic questionnaire covered gender (they identified themselves as a man or woman), age, sexual orientation (gay, lesbian, bisexual, heterosexual), time spent on social media sites (in hours), relationship status, and the current (or most recent) relationship duration. Regarding the time spent on social media sites, the participants reported between 1 h and 8 h a day. All of these data were self-reported.

The *Attitudes Toward Infidelity Scale* (*ATIS)* [70] measures 12 attitudes toward the attitude of the participant toward infidelity in general. The scale checks on a 7-point rating scale (1 = *strong disagreement*, 7 = *strong agreement*) to what extent the individual agrees or disagrees with the mentioned assertions. The scale contains items such as: “*I would have an affair if I knew my significant other would never find out*” or “*Infidelity is morally wrong in all circumstances regardless of the situation*”. High scores indicate a positive attitude toward infidelity and low scores indicate a negative attitude toward infidelity. The coefficient of internal consistency for this study is α= 0.77.

The *Problematic Internet Questionnaire Short Form (PIUQ-SF-6)* [71] measures, by use of 6 items, thoughts and behaviors related to internet use and its effects on the psycho-social health of the individual (“*How often do you spend time online when you’d rather sleep?*”; “*How often do you feel that you should decrease the amount of time spent online?*”). All items were answered on a 5-point rating scale (1 = *never* to 5 = *always*) and the score on the scale ranged from 6 to 30. The problematic use of the internet increases as the score increases. The internal consistency factor is α= 0.77.

*The Couples Satisfaction Index* [72] is a 16-item scale that measures the degree of satisfaction in the current relationship. Some questions are rated on a 7-point rating scale, where 0 means extremely unhappy and 6 means perfect, and other items ask participants to indicate the extent of agreement or disagreement in couple (“*Amount of time spent together*”) from 5 (*always agree*) to 0 (*always disagree*). There are other types of items that ask participants to indicate frequency of some thoughts (“*In general, how often do you think that things between you and your partner are going well?*”) from 5 (*all the time*) to 0 (*Never*), and other types of items in which individuals have to respond from “not at all true” to “completely true” on a 5-point rating scale (“*I feel that I can confide in my partner about virtually anything*”). Higher scores indicate a high level of dyadic satisfaction, and lower scores indicate a low level of dyadic satisfaction. For this study, the Alpha Chronbach is α= 0.95.

*Social Media Infidelity-Related Behaviors (SMIRB)* [5] is a scale of 7 items that measures IR behaviors on social media sites. These IR behaviors might involve those who are unfaithful (such as feeling uncomfortable, hiding information/secrets, forming emotional connections with other people, and message conversations with former partners). The scale contains items like: “*I would feel uncomfortable if my spouse/partner read my chats, comments, and messages to others on social networking sites*” and “*I sometimes hide the things I say to others online from my spouse/partner*”. Scoring is a 6-point rating scale (1= *strongly disagree*; 6 = *strongly agree*). Higher scores indicate a greater tendency to engage in IR behaviors on social media sites and lower scores indicate the absence of these IR behaviors or rare occurrences. The internal consistency measured in this study shows an index of α= 0.79.

### 2.3. Statistical Analysis

Statistical analyses were performed using SPSS Version 24. Shapiro-Wilk test for goodness-of-fit was used to assess the normality of the distribution. Continuous variables that were not normally distributed (i.e., the distribution of scores measuring dyadic satisfaction, attitudes toward infidelity, and IR behaviors on social media sites) were transformed using the fractional rank method. Gender and sexual orientation were categorized into dummy variables (1: men, 0: women; 1: sexual minority individuals, 0: heterosexual). 

## 3. Results

### 3.1. Descriptive Analyses and Group Comparison

Statistical analyses for the transformed variables were based on transformed values, whereas descriptive statistics were based on original values. Table 2 displays the descriptive statistics.

Data analysis showed there were no significant differences between GL (*M* = 17.78, *SD* = 5.91) and heterosexual individuals (*M* = 14.55, *SD* = 6.58) in engaging in social media IR behaviors, *t*(119) = −2.64, *p* >0.05.

### 3.2. Correlations between IR Behaviors on Social Media Sites and Variables

Pearson correlation analyses were separately performed for GL and heterosexual participants (see Table 3). In GL participants, an inspection of the correlation matrix did not show a correlation between IR behaviors and other variables. In heterosexual participants, online IR behaviors were positively related to time spent on the internet (*r* = 0.30, *p* = 0.006), and negatively correlated with dyadic satisfaction (*r* = −0.36, *p* = 0.001), showing a medium effect size. 

### 3.3. Analyses of Variance ANOVA

A 2 × 2 between-subjects design using sexual orientation (GL and heterosexual) and attitudes toward infidelity (positive attitude and negative attitude) evaluated the degree to which participants experienced IR behaviors on social media sites. While the main effect of sexual orientation *F*(1, 120) = 1.55, *p* > 0.05 was not significant, the main effect of attitudes toward infidelity was significant, *F*(1, 120) = 6.74, *p* < 0.05, *η_p_*^2^ = 0.05. The interaction effect between sexual orientation and attitude toward infidelity was not significant, *F*(1, 120) = 0.01, *p* > 0.05. Means and standard deviations are presented in Table 4. 

Contrasts analysis was conducted using differences. A significant difference between participants with a positive attitude toward infidelity and a negative attitude toward infidelity was found at *p* < 0.05 (*M* = 3.43, *SD =* 1.32), which means that those with a positive attitude (*M* = 17.94, *SD* = 0.98) were more likely to engage in IR behaviors on social media sites than those with a negative attitude (*M* = 14.51, *SD* = 0.87). The contrast differences are presented in Table 5.

A 2 × 2 (sexual orientation × low vs. high dyadic satisfaction) analysis of variance (ANOVA) showed a significant main effect of sexual orientation *F*(1, 120) = 4.77, *p* < 0.05, η^2^partial = 0.031, which means GL participants (*M* = 17.55, *SD* = 1.02) were more likely to report dyadic satisfaction compared to non-GL individuals (*M* = 14.82, *SD* = 0.71) and this difference was significant *p* < 0.05. However, no interaction effect of the two variables was found and no main effect of the variable dyadic satisfaction was significant *F*(1, 120) = 0.10, *p* > 0.05, which means that the effect of sexual orientation on IR behaviors does not appear dependent on dyadic satisfaction.

A 2 × 2 ANOVA was conducted in order to explore the interaction between sexual orientation (sexual minority individuals vs. heterosexual) and problematic internet usage (non-problematic internet use vs. problematic internet use) among IR behaviors on social media sites. The results showed a significant main effect of sexual orientation *F*(1, 120) = 7.71, *p* < 0.05, *η_p_*^2^ = 0.062 on IR behaviors on social media sites. A significant main effect of problematic internet use *F*(1, 120) = 6.75, *p* < 0.05, η^2^ partial = 0.055 was also found; no significant interaction effect was found *F*(1, 120) = 0.07, *p* = 0.78. 

Analyses of variance showed no significant main effect of gender or interaction effect between sexual orientation and gender among any of the study variables (*p* > 0.05).

## 4. Discussion

This cross-sectional study examined the relationship between IR behavior on social media sites, dyadic satisfaction, attitudes toward infidelity, and problematic internet usage, for GL and non-GL unmarried individuals in a post-communist socio-cultural context. Specifically, we tested whether engaging in IR behaviors on social media sites, attitude toward infidelity, problematic internet usage, and dyadic satisfaction differ as a function of sexual orientation and whether engaging in IR behaviors on social media sites is related to attitudes toward infidelity, problematic internet usage, and dyadic satisfaction, among unmarried GL and heterosexual individuals.

Our results did not show significant differences between GL and heterosexual individuals regarding IR behaviors. Previous studies regarding infidelity have reported that GL and heterosexual individuals were similar in the type of infidelity that would upset them the most, sexual or emotional [73], and they reported similar experiences of cognitive and emotional jealousy [74]. Other studies examining GL and heterosexual individuals have shown that there are differences in the type of infidelity, with heterosexual men being more upset by sexual infidelity but less upset by emotional infidelity than heterosexual women and GL participants [68]. Furthermore, GL individuals responded with less intense jealousy to scenarios describing a partner having sex with someone else, in comparison to non-GL individuals [67]. However, our results indicating that gender and sexual orientation did not significantly interact regarding study variables are in line with previous studies [75].

Results may be explained by the fact that in a previous study that measured social media IR behaviors in a group of people with a non-GL orientation, only a small percentage of married/cohabiting participants were involved in social media IR behaviors [5]. However, compared to this, the present research did not include married participants, and the comparison was made between GL and heterosexual individuals. According to the Ecological perspective of the Couple and Family Technology Framework [11], we concluded that, for GL individuals, the virtual space is not perceived as more threatening for the relationship compared to non-GL individuals, thus, the implication in social media IR behaviors is similar for both groups.

The results indicated a main effect of attitudes toward infidelity among IR behaviors on social network sites. This finding is in line with previous research which reported that personal norms are an important factor in deciding whether to engage in infidelity behaviour [32]. Other researchers reported that permissive sexual attitudes have been identified as a possible predictor of infidelity, while one study of young adults suggested that positive attitudes toward infidelity and lower commitment were significantly associated with infidelity behaviors [43]. Our results may be explained also in terms of attitude, which represents a person’s predisposition to interpret something in a favorable or unfavorable manner [76]. Thus, if a person perceives IR behaviors as favorable, he will get involved in them, and if he perceives them as unfavorable for him and for the relationship, he will not get involved.

A significant main effect of sexual orientation on IR behaviors on social media sites was found. However, no interaction effect was found between sexual orientation and dyadic satisfaction. Previous studies on non-LG married individuals reported that greater IR behavior was related to lower relationship satisfaction [5]. One possible explanation is that married/cohabiting couples who were less satisfied were more likely to report engaging in IR behaviors [5]. In the current study, we eliminated married couples, our sample being formed only from individuals who were or were not in an unofficial romantic relationship. The Interpersonal Exchange Model of Sexual Satisfaction [77] postulated that, in same-sex couples, sexual satisfaction is negatively associated with internalized homophobia, the number of sexual costs, anxiety, and avoidance. All these variables may be explanatory for the lack of a main effect and interaction effect between dyadic satisfaction, sexual orientation, and implication in IR behaviors. Another possible explanation is the influence of sociosexuality, an individual variable that measures a person’s level of comfort when engaging in sexual behaviors outside of the couple’s relationship [78]. Twenge et al. [79] reported that general levels of sociosexuality are less and less restricted, which indicates greater comfort for people when engaging in extradyadic relationships. Moreover, the same authors report that generations born later in the twentieth century showed a less restrictive attitude toward casual sex, compared to people born earlier in the twentieth century.

Our analyses showed that problematic internet usage has a main effect on online IR behaviors, but not the length of time spent on social media sites. Specifically, participants who reported problematic internet usage were more likely to engage in IR behaviors on social media sites. Previous studies have shown that 42% of individuals who excessively used the internet were involved in a romantic relationship online even though they had an offline partner [30]. Thus, we concluded that the more problematic internet usage, the more favorable the probability to engage in IR behaviors, independent of sexual orientation.

Our study presents data from an underrepresented cultural context. A potential explanation for these findings consists in the fact the current study was conducted in Romania, which is among the most intolerant European countries with respect to sexual minority individuals [80]. According to the European Values Survey 1999/2000 data set which investigated culture-level determinants of negative attitudes toward sexual minority individuals, 77% of Romanian participants believed that homosexuality is not justifiable and 65.2% reported that they do not want to have a sexual minority individual as a neighbor [80]. Moreover, examining sexuality-related attitudinal patterns across 32 European countries, Lottes and Alkula [81] found that most post-communist countries were distinguished from the rest of Europe by low justifications of homosexuality. Therefore, these individuals may encounter discrimination and stigma based on their sexual orientation if they openly identify themselves as being gay in a society that devalues their sexuality. Social media sites facilitate secure and private interpersonal communication between sexual minority partners in this social climate, providing the opportunity for and engagement in romantic relationships and IR behaviors.

## 5. Limitations and Implications for Future Research

This study has multiple limitations. A first limit of the present research that should be addressed in future research is related to the impossibility of controlling the multitude of variables that influence online behavior. For example, we do not control the motivations that lead to certain online behaviors that might be associated with infidelity (friendship and conversation with former partners, offering “likes”, photo comments). Our findings may also be influenced by the number of GL individuals. We were unable to recruit more GL individuals because of the very limited LGBT communities in the country, as well as the reluctance of these people to participate in the research, despite ensuring anonymity. Furthermore, our findings would benefit from additional information that was not collected in the demographic questionnaire. For example, we do not know the participants’ level of education.

Future research should focus on other relationships and personal characteristics, such as the history of infidelity. As Sagarin et al. [82] reported, men who were victims of infidelity were more affected by sexual infidelity. Women who had cheated on their partner in the past indicated sexual infidelity as more annoying, compared to women who do not have a history of infidelity. Furthermore, in terms of dyadic satisfaction, future research may consider measuring couple communication. Researchers suggest that couples who experience infidelity show less positive premarital interactions and more negative and invalidating interactions [83]. Additionally, future studies may consider different types of romantic relationship agreements (e.g., open relationships, polyamory) and online infidelity. For example, it would be interesting to study IR behaviors on social media sites in consensual nonmonogamy among sexual minorities.

Future research may also address similar studies in different cultural contexts, as contradictory conclusions from previous studies may be due to sociocultural differences. Moreover, a modified scale should be used depending on the context of the research and culture [84]. On the one hand, a further improvement for future studies might offer better representativeness of LGBT populations since most of the literature on LGBT issues has focused on the first two letters of the acronym, making bisexual and trans* people invisible [85]. On the other hand, in Romania and in many other societies, people from LGBT communities are not so accepted, and sexual prejudice and stereotyping are still widespread, making it difficult to recruit a large number of LGBT people [86]. It is possible to obtain other results in the context of conducting the study in a country that allows the free expression of these groups.

On a related note, future studies might include a measure of internalized sexual stigma, which can influence IR behaviors and the satisfaction and wellbeing of same-sex couples [55,56,57]. This would allow a clearer picture and a better comprehension of the phenomenon, by emerging potential interactive roles with the variables investigated.

In conclusion, we firmly believe that studies like this might help foster the wellbeing of same-sex couples by making them aware of the processes and challenges they face to achieve couple wellbeing and satisfaction in a stigmatizing, hetero-normative, and heterosexist society. Potential relationship education programs, which include issues related to heteronormativity internalized sexual stigma and stereotypes about same-sex couples, might be useful for couples formed by people in the sexual minority, equipping them with adequate resources to face challenges that can hinder their wellbeing and satisfaction as a couple [52].

## 6. Conclusions

Previous studies have shown that similar relationship characteristics might influence both offline and online IR behaviors in married/cohabiting heterosexual individuals [5]. The present study offers an extension to prior work, showing that online IR behaviors were not influenced by similar variables in GL and non-GL unmarried individuals. Our findings add to the existing literature on the correlates of online IR behaviors among same-sex couples and opposite-sex couples’ relationships. Because systems of oppression and privilege have impacted human sexual desire experiences, additional research is needed, especially from “gay-friendly” social climates [87], in order to gain a better understanding of the dynamics of all human relationships. Mental health professionals might find these findings helpful for understanding the differences and similarities in approaching GL and heterosexual couples when confronted with IR behaviors online and problematic internet usage.

## Figures and Tables

**Table 1:** Sociodemographic characteristics of the sample.

Variables	M	*SD*	N
Age	23.53	4.7	
Gender			
Men			65
Women			56
Relationship duration (months)	31.64	31.48	
Sexual orientation	1.33	0.47	
Heterosexual			81
Gay			30
Lesbian			10
Social media sites account			
Facebook			10
Facebook, Instagram			86
Facebook, other social media accounts			25

**Table 2:** Descriptive statistics (means and standard deviations) from non-transformed distribution.

Variable	Gay and Lesbian (n = 40)		Heterosexuals (n = 81)	
	Men (n = 30)	Women (n =10)	Men (n = 35)	Women (n =46)
	M *(SD)*	M *(SD)*	M *(SD)*	M *(SD)*
Age	24.13 (6.24)	20.7 (2.31)	23.46 (4.88)	23.8 (3.55)
Relationship duration (months)	31.83 (39.29)	23.9 (30.88)	29.66 (25.31)	34.7 (30.78)
Time spent on social media sites (hours)	3.83 (1.84)	3.5 (1.78)	4.11 (2.05)	3.7 (2.08)
PIUQ-SF	16.46 (4.61)	17.9 (5.54)	16.51 (5.41)	17.32 (4.81)
CSI	54.71 (13.58)	62.53 (16.23)	64.88 (14.77)	60.7 (15.27)
SMIRB	18.11 (6.16)	16.43 (4.72)	14.71 (7.07)	14.13 (6.23)
ATIS	32.03 (9.07)	27.49 (8.6)	25.92 (9.82)	19.96 (10.58)

Note. PIUQ-SF = Problematic Internet Questionnaire Short Form; CSI = The Couples Satisfaction Index; SMIRB = Social Media Infidelity-Related Behaviors; ATIS = Attitudes Toward Infidelity Scale.

**Table 3:** Bivariate correlations between study variables for gay and lesbian (above) individuals and separately for heterosexual individuals (below).

Variable	1.	2.	3.	4.	5.	6.	7.	8.
1. Age	1	−0.29	−0.25	0.13	0.02	−0.13	0.08	−0.006
2. Gender	0.81	1	0.01	−0.10	0.09	0.14	−0.06	−0.16
3. Relationship duration (months)	−0.05	0.11	1	0.10	−0.20	0.28	0.01	−0.09
4. Time spent on social media (hours)	0.16	−0.9	0.11	1	0.15	0.04	0.26	−0.18
5. PIUQ-SF	−0.18	0.08	0.13	−0.08	1	−0.31 **	0.004	−0.21
6. CSI	−0.26 *	−0.14	0.06	−0.11	0.05	1	−0.12	−0.15
7. SMIRB	0.09	−0.02	0.15	0.86	0.30 **	−0.36 **	1	0.25
8. ATIS	0.06	−0.29 **	−0.08	0.02	−0.08	−0.04	0.33 **	1

Note. * *p* < 0.01, ** *p* < 0.001 PIUQ-SF = Problematic Internet Questionnaire Short Form; CSI = The Couples Satisfaction Index; SMIRB = Social Media Infidelity-Related Behaviors; ATIS = Attitudes Toward Infidelity Scale.

**Table 4:** Means and standard deviations for sexual orientation, attitude toward infidelity, couple satisfaction, and problematic internet use.

	Sexual Orientation			
	Gay and Lesbian		Heterosexual	
	*M*	*SD*	*M*	*SD*
ATIS				
Negative attitude	15.42	3.58	13.60	6.22
Positive attitude	18.69	6.54	17.20	6.88
CSI				
Low couple satisfaction	18.34	6.24	16.01	5.98
High couple satisfaction	16.76	5.57	13.63	6.86
PIUQ				
Non-problematic internet use	16.57	5.74	12.86	6.02
Problematic internet use	19.41	6.06	16.35	6.70

Note. ATIS = Attitudes Toward Infidelity Scale; CSI = The Couples Satisfaction Index; PIUQ = Problematic Internet Questionnaire Short Form.

**Table 5:** Contrast differences for sexual orientation, attitude toward infidelity, couple satisfaction, and problematic internet use on social media infidelity-related behaviors.

Variable	Contrast Estimate	*p*
*Sexual orientation*	−1.648	0.215
*ATIS*	3.432	0.011
*Sexual orientation*	−2.730	0.031
*CSI*	−1.982	0.115
*Sexual orientation*	−3.384	0.006
*PIUQ-SF*	3.168	0.011

Note. ATIS = Attitudes Toward Infidelity Scale; CSI = The Couples Satisfaction Index; PIUQ-SF = Problematic Internet Questionnaire Short Form.

## Data Availability

The data that support the findings of this study are available from the corresponding author upon reasonable request.
